# A Digital Outpatient Service With a Mobile App for Tailored Care and Health Literacy in Adults With Long-Term Health Service Needs: Multicenter Nonrandomized Controlled Trial

**DOI:** 10.2196/60343

**Published:** 2025-04-28

**Authors:** Heidi Holmen, Are Martin Holm, Ragnhild Sørum Falk, Thomas Karsten Kilvær, Tone Marte Ljosaa, Christopher Ekholdt, Erik Fosse

**Affiliations:** 1 Intervention Centre Division of Technology and Innovation Oslo University Hospital Oslo Norway; 2 Department of Nursing and Health Promotion Faculty of Health Sciences Oslo Metropolitan University Oslo Norway; 3 Department of Respiratory Diseases Division of Cardiovascular and Pulmonary Diseases Oslo University Hospital Oslo Norway; 4 Institute for Clinical Medicine Faculty of Medicine University of Oslo Oslo Norway; 5 Oslo Centre for Biostatistics and Epidemiology Oslo University Hospital Oslo Norway; 6 Department of Cancer University Hospital of Northern Norway Tromsø Norway; 7 Institute for Clinical Medicine Faculty of Medicine The Arctic University of Norway Tromsø Norway; 8 Department of Pain Management and Research Division of Emergencies and Critical Care Oslo University Hospital Oslo Norway

**Keywords:** outpatient, digital solution, mobile health, mHealth, remote monitoring, health literacy, self-monitoring, patient-reported outcome measures, cancer, complex pain, epilepsy, interstitial lung disease

## Abstract

**Background:**

Patients with long-term health needs are often expected to actively participate in outpatient care, assuming that they have appropriate health literacy and digital health literacy. However, the association between participation in a digital outpatient service and health literacy remain unclear.

**Objective:**

This study aims to evaluate whether digital outpatient care for 6 months improved health literacy, health-related quality of life (HRQoL), digital/eHealth literacy, and the use of health care services compared with usual care.

**Methods:**

We conducted a multicenter nonrandomized trial with 1 intervention arm and 1 control arm. Patients aged ≥18 years receiving outpatient care in the pain, lung, neurology, or cancer departments at 2 Norwegian university hospitals were allocated in a 1:2 ratio, favoring the intervention arm. The intervention arm received digital outpatient care using tailored patient-reported outcome measures, self-monitoring, and chats for timely contact with the outpatient clinic. Patient responses were assessed by health care workers via a dashboard with a traffic light system to draw attention to the most urgent reports. The control arm received usual care. The data were collected at baseline and after 3 and 6 months. The primary outcome was the change in health literacy according to the Health Literacy Questionnaire domain *understanding health information well enough to know what to do* from baseline to 6 months. The mean difference in change between the 2 treatment arms was the effect measure. The secondary outcomes were additional domains from the Health Literacy Questionnaire, digital/eHealth literacy, HRQoL, acceptability of the digital intervention, and health service use.

**Results:**

Overall, 162 patients were recruited, 55 (34%) in the control arm and 107 (66%) in the intervention arm, with a 17.3% attrition rate after 6 months. There was no statistically significant difference in the primary outcome, “understanding health information well enough to know what to do,” between the arms at 6 months (mean difference –0.05, 95% CI –0.20 to 0.10; *P*=.53). After 3 months, the health literacy domains *actively managing my own health* (–0.15, 95% CI –0.30 to –0.00; *P*=.048) and *understanding health information well enough to know what to do* (–0.17, 95% CI –0.34 to –0.00; *P*=.03), as well as both physical (–3.29, 95% CI –5.62 to –0.96; *P*=.006) and mental HRQoL (–3.08, 95% CI –5.64 to –0.52; *P*=.02), improved in the digital outpatient intervention arm compared with the control arm.

**Conclusions:**

This study explored digital outpatient care. Although no statistical differences were observed in patients’ health literacy after 6 months, our data indicate an improvement in health literacy domains and HRQoL at 3 months. The participants reported high satisfaction with the digital outpatient care intervention, and our findings highlight the potential of digital interventions in outpatient care.

**Trial Registration:**

ClinicalTrials.gov NCT05068869; https://clinicaltrials.gov/ct2/show/NCT05068869

**International Registered Report Identifier (IRRID):**

RR2-10.2196/46649

## Introduction

### Background

The implementation of digital health services in specialized health care services necessitates an understanding of the purpose and use of digital services by both patients and health care workers [1,2]. The potential of digital health solutions is recognized by health authorities and stakeholders; however, despite systematic reviews exploring digital health services, the impact of digital solutions on resource use and patient outcomes, such as symptoms, self-management, quality of life, digital health literacy, and satisfaction, remains uncertain [2-9]. Digital services may improve resource use and provide patients with supplementary and understandable health information; the potential benefit and relevance of digital health solutions increase as medical advancements extend life expectancy and as patients with chronic or long-term conditions in need of frequent and repeated care use a large proportion of the available consultations [9,10]. Exploring how to exploit this potential is crucial, as the increase in patients, combined with constrained health resources, presents obstacles to the sustainability of current health services [11]. Furthermore, reductions in hospital beds may increase the need for more outpatient consultations, which is an arena where digital health services have the potential to aid timely and appropriate care.

To fulfill the potential of digital health solutions, attaining a certain level of health literacy is pivotal because health literacy positively influences both self-management and health-related decisions [2,12-15]. Health literacy is often defined, according to Nutbeam [16], as “the cognitive and social skills that determine the motivation and ability of individuals to gain access to, understand, and use information in ways which promote and maintain good health.” Consequently, digital health literacy concerns the use of digital solutions for promoting and maintaining good health [2]. Low health literacy is associated with poor health outcomes and less digital solution use [4,9,14,17,18], while improved health literacy may enhance self-management and the benefits of digital health solutions [1]. Thus, in accordance with a conceptual model proposing how health literacy is affecting interaction with services and health care workers and outcomes [15], we argue that digital health interventions specifically allowing for more patient-centered and flexible care might support patients’ health literacy and aid in their self-management [19]. However, there is a lack of research exploring the role of health literacy in digital health interventions, although the capacity to benefit from digital health solutions and improve self-management is likely to be enhanced by improved health literacy [2,9]. In addition, there exists a similar gap in knowledge on the relation between digital health literacy and digital health interventions [9].

Digital health solutions in outpatient care typically enable the subjective reporting of health parameters through patient-reported outcome (PRO) measures. PRO measures may aid in self-management and communication between patients and health care workers [20]. Digital engagement, self-monitoring, and data sharing offer advantages for patients; however, whether a digital health solution is meaningful depends on its usability, clinical relevance, and convenience, along with whether it has an evidence-based design [9,20-22]. Recent studies indicate that digital solutions in outpatient care can prevent complications, foster patient engagement, and boost confidence and autonomy [23-26]. However, research on multicomponent digital solutions encompassing PRO measures, asynchronous messaging, remote monitoring, patient notifications, and video consultations is limited. Positive effects have been noted in cancer treatment [24], musculoskeletal pain disorders management [25], and home monitoring for interstitial lung disease (ILD) [23]. Mobile apps for patients with epilepsy have shown promising results; however, the value of patients’ collaborating with health care workers remains unclear [26]. Challenges persist, such as integrating electronic health records and standardizing data for registry use. In summary, studies exploring the implementation and impacts of digital health solutions in outpatient care are warranted.

### Objectives

The objective of this study was to evaluate whether digital outpatient care for 6 months resulted in improved health literacy, health-related quality of life (HRQoL), digital/eHealth literacy, and the use of health care services compared with usual care.

Specifically, we hypothesized that digital outpatient care would be associated with a favorable increase in patients’ ability to understand health information well enough to know what to do. By enabling digital access to services, facilitating patient reports and self-monitoring, and promoting interaction with both the digital solution and health care workers, our intervention reinforces a conceptual model for associations between health literacy and health outcomes [15,19].

## Methods

### Trial Design

We conducted a prospective, multicenter, nonrandomized controlled trial with 2 arms and a 6-month follow-up, using a longitudinal design with 3 assessment points: baseline, 3 months, and 6 months (from September 2021 to June 2023). The control arm received the usual care, while the intervention arm received the digital outpatient care intervention. The trial was registered in Clinical Trials and the International Registered Report Identifier (DERR1-10.2196/46649). The evaluation was based on elements of the method for assessment of telemedicine [27], and our study was reported according to Strengthening the Reporting of Observational Studies in Epidemiology (STROBE) checklist [28], while the PRO measures were reported according to the Standard Protocol Items: Recommendations for Interventional Trial (SPIRIT) PRO Extension [29]. The development of the intervention and study protocol have been reported in detail elsewhere [19].

### Participants and Setting

Patients were recruited from the department of respiratory diseases, the department of neurology, and the department of pain management and research at the Oslo University Hospital as well as the department of cancer at the University Hospital of North Norway. Eligible patients were required to be aged ≥18 years, based at home, and capable of completing questionnaires in Norwegian. Inclusion criteria were applied to patients who were newly diagnosed or referred or to those with a diagnostic history in outpatient care. Patients with cancer had to receive active treatment, have a life expectancy of at least 6 months, and have an expected need for services beyond 6 months. Patients with ILD were eligible unless they had a very low degree of function with cognitive impairment. Patients with epilepsy were included unless they had complex causes, severe comorbidities, or ongoing assessments or treatments. Patients with long-term pain were eligible if they underwent drug testing and self-administered their medication and were not eligible if they had comorbidities that directly affected drug adjustment, had cognitive impairment, or did not live at home.

Eligible patients were identified through consultations or patient lists provided by health care workers from each department. The patients received project information before written consent was obtained. At the 3- and 6-month follow-ups, the patients received either an SMS text message or an email invitation to complete their follow-up questionnaire. Consent and all questionnaire responses were provided through the encrypted version of the web-based questionnaire service Nettskjema (University of Oslo) [30]. The data were stored using a service for sensitive data (TSD), complying with the Norwegian Personal Data Act and Health Research Act. This service required a governmental ID portal for log-in, hence ensuring secure data harvesting. In the event of no response after 1 week, a reminder was sent, followed by a phone call 1 week later for pending questionnaires.

Consent and baseline questionnaire completion were prerequisites for a fulfilled allocation for both the control and intervention participants. In addition, this completion of consent and baseline questionnaire was required to access the digital intervention in the intervention arm to reduce contamination in the baseline questionnaire responses.

Participants recruited in the control arm received usual care per routine at the outpatient clinic where they received care. This included the possibility of contacting outpatient departments by telephone during office hours and regular in-person consultations.

### Intervention

The details of the development and tailoring of the intervention have been previously presented [19]. The Dignio Connected Care platform facilitated timely, personalized contact between patients and health care workers [31]. This platform, including the Dignio Prevent system for health care workers and the MyDignio patient app, is Conformité Européene marked, compliant with privacy regulations, and successfully applied in various global clinical settings. Customizable to individual patient needs, components were added for personalized care, with interface examples provided in Multimedia Appendix 1.

Health care workers from each department assigned tasks to patients in Dignio Prevent and set thresholds for individual patients using a traffic light model. Notifications alerted health care workers if measures deviated, such as patients reporting an increase in pain and side effects, or if the task went undone. The patients received reminders through the MyDignio app and an SMS text message if the task remained undone. Two-way messaging enabled asynchronous communication, allowing patients to ask questions or provide relevant information to their health care workers; likewise, health care workers could respond in due time based on the urgency of the message and their workload. For instance, treatment plans were shared with the patients through the chat messaging function, allowing them to always have access to their current plan. MyDignio app’s information page, which was updatable to health care workers, allowed for individualized updates and templates for multiple patients if needed.

The intervention’s core focus across all 4 departments was improving outpatient service accessibility. The app allowed patients to engage with PRO measures, self-monitor relevant parameters, and communicate asynchronously with health care workers (Table 1). Designated staff assessed the responses, allowing for flexible patient care, which is a strategy aligned with a conceptual model linking health literacy to service access, use, interactions between patient and health care worker, and self-management [15,19].

**Table 1:** Patient-reported components used in the digital outpatient service intervention [19].

Dignio component			Short description		Department using the component in its intervention
**PRO^a^ measures**					
	Self-reported measures	Standardized and individualized PRO measures with numerical scales, single and multiple-choice answers, and free text		Cancer department: ESAS-r^b^ [32] and ECOG^c^ scales were used for assessing functional level [33]. The frequency of administration was per individual needs. Lung department: K-BILD^d^ questionnaire [34] was used to assess self-reported health every 12th week, and items on side effects (vomiting, rash, dizziness, abdominal pain, or diarrhea) were administered every fourth week. Neurology department: PRO-EPI^e^, which is a multidimensional epilepsy PRO measure with items on seizures, medication, living with epilepsy, and the need for health services developed by clinicians and the Norwegian Epilepsy Network for health care workers [35], was administered every 11th week. Pain department: scale to assess pain intensity (NRS^f^ score of 0-10), location, analgesics’ side effects [eg, constipation, sweat, and anxiety]), compliance with current treatment, and the need for health services were administered every 3 days.	
**Physiological measures**					
	Blood pressure	BYOD^g^		Cancer department, on clinical indication	
	Body temperature	BYOD		Cancer department, on clinical indication	
	Body weight	BYOD		Lung department, weekly	
	Spirometry values	Bluetooth device		Lung department, weekly	
	Oxygen saturation	Bluetooth device		Lung department, weekly	
	Pulse	BYOD		Lung department, weekly	
**Patient-reported free text**					
	Free-text messages	Free-text messages sent from the patient to the service		All departments	

^a^PRO: patient-reported outcome.

^b^ESAS-r: Edmonton Symptom Assessment System Revised.

^c^ECOG: Eastern Cooperative Oncology Group.

^d^K-BILD: King’s Brief Interstitial Lung Disease.

^e^PRO-EPI: patient-reported outcome–epilepsy.

^f^NRS: Numeric Rating Scale.

^g^BYOD: bring your own device.

Patients in the intervention arm were briefly introduced to the app, but no extensive training was provided because the MyDignio app was set to be intuitive. Those using spirometers were instructed to ensure the correct technique and synchronization with the MyDignio app. The intervention arm participants could contact health care workers or researchers for technical assistance.

Most health care workers were involved in developing the intervention; therefore, they were familiar with the platform. The training on interpreting patient-reported scores was department specific. The designated health care workers received compensation for 10% to 20% of their time.

### Measures

#### Data Collection

All self-reported data were collected through questionnaires sent to the participants through the Pretty Good Privacy–encrypted version of the University of Oslo web-questionnaire service Nettskjema [30], which uses a governmental ID portal for log-in and allows secure data harvesting, previously described in our protocol [19]. A secure, personal link was sent to the participants’ email or mobile phone, with 1 reminder in the case of unanswered questionnaires after 6 to 7 days, followed by a phone call if they still did not respond. The participants self-reported the demographic data at baseline, while clinical measures were collected from their medical records at baseline and 6-month follow-up. Data on the use of digital service intervention and health services were collected from medical records and the digital platform after 6 months. The primary outcome, domain 9 of the Health Literacy Questionnaire (HLQ), *understanding health information well enough to know what to do*, and secondary outcomes comprising domains 1, 2, 3, and 6 of HLQ; the eHealth Literacy Questionnaire (eHLQ); and RAND-12 health survey questionnaire were self-reported at baseline and the 3- and 6-month follow-ups. General satisfaction and satisfaction with the digital intervention were self-reported after 3- and 6-month follow-ups.

#### Demographics

Demographic characteristics included age, sex, education, employment status, marital status, and digital skills. The full range of demographic data and their scores has been reported elsewhere [19].

#### Clinical Measures

Clinical information included the primary diagnosis and its duration, medication, and comorbidities. Medication, contact with health care services, and hospitalization during the 6 months of the trial were recorded at the 6-month follow-up. Lifestyle habits, including smoking, the use of snuff, and alcohol consumption, were self-reported at baseline. History of previous infection with COVID-19 and fear of COVID-19 were self-reported and collected at all 3 time points. The fear of COVID-19 was reported only among those without a previous infection of the virus.

#### Use of the Digital Service Intervention

Throughout the intervention period, the use of the digital outpatient service was recorded. This included data on the number of PRO measure responses, the number of chat messages sent and received by the patients, and the number of physiological measures uploaded. A total score of intervention use containing all responses from the patient and interactions with health care workers was calculated. The total number was dichotomized into low use and high use based on the expected use per department (Table 1). Low-use patients responded to <30% of the expected PRO measures.

#### Primary Outcome

The primary outcome was domain 9 of the HLQ, *understanding health information well enough to know what to do* [36]. This domain includes 5 items covering health-related competencies essential for using a digital service, such as fundamental reading and comprehension of health information, adherence to health care workers’ instructions, and the capability to accurately complete forms [36,37]. This domain was self-reported at all 3 time points using a 5-point scoring scale, “cannot do or always difficult,” “usually difficult,” “sometimes difficult,” “usually easy,” and “always easy,” where a higher score indicates a higher level of health literacy. The HLQ is a validated measure translated into Norwegian and adapted to the Norwegian context [37]. Unfortunately, 1 of the 5 items, item 12, “read and understand written health information*,*” was not collected because of an administrative error. Thus, the mean score was based on the remaining 4 items. Cronbach α was 0.72 for domain 9.

#### Secondary Outcomes

Health literacy was assessed using an additional 4 of the 9 domains of the HLQ, including domain 1, *feeling understood and supported by health care providers* (4 items); domain 2, *having sufficient information to manage my own health* (4 items); domain 3, *actively managing my own health* (5 items); and domain 6, *ability to actively engage with health care providers* (5 items) [36]. Cronbach α was satisfactory for all domains (domain 1: Cronbach α=0.79; domain 2: Cronbach α=0.84; domain 3: Cronbach α=0.83; and domain 6: Cronbach α=0.90).

Digital/eHealth literacy was measured using all 7 domains of the eHLQ [38], including domain 1, *using technology to process health information* (5 items); domain 2, *understanding health concepts and language* (5 items); domain 3, *ability to actively engage with digital services* (5 items); domain 4, *feel safe and in control* (5 items); domain 5, *motivated to engage with digital services* (5 items); domain 6, *access to digital services that work* (6 items); and domain 7, *digital services that suit individual needs* (4 items). Higher scores indicate higher levels of digital/eHealth literacy. The eHLQ has been validated and translated into Norwegian [39]. Cronbach α was satisfactory for all domains in this study (domain 1: Cronbach α=0.82; domain 2: Cronbach α=0.75; domain 3: Cronbach α=0.85; domain 4: Cronbach α=0.85; domain 5: Cronbach α=0.79; domain 6: Cronbach α=0.74; and domain 7: Cronbach α=0.78).

HRQoL was assessed using the RAND-12 questionnaire [40,41], which contains 12 items, informing 1 physical and 1 mental component score, where a higher standardized score indicates better HRQoL [42]. Cronbach α was 0.86 for the physical score and 0.79 for the mental score in the sample.

General patient satisfaction was assessed among all participants, encompassing the following 3 items: satisfaction with treatment, benefit from treatment, and whether a digital outpatient service would affect safety.

Satisfaction related to the intervention was only assessed in the intervention arm and measured using the Service User Technology Acceptability Questionnaire (SUTAQ), which comprises 22 items with a 6-point Likert scale [43]. The SUTAQ contains six domains, including domain 1, *enhanced care* (5 items); domain 2, *increased accessibility* (4 items); domain 3, *privacy and discomfort* (4 items); domain 4, *care personnel concerns* (3 items); domain 5, *kit as a substitute* (3 items); and domain 6, *satisfaction* (3 items). For domains 1, 5, and 6, a higher score indicates greater satisfaction, while for domains 3 and 4, the opposite is true, where a higher score represents a concern. Cronbach α was satisfactory (Cronbach α≥0.7) for 4 of the 6 domains in this study, including domain 1 (Cronbach α=0.87), domain 2 (Cronbach α=0.84), domain 3 (Cronbach α=0.73), domain 4 (Cronbach α=0.62), domain 5 (Cronbach α=0.46), and domain 6 (Cronbach α=0.89). The SUTAQ was previously translated and applied in a Norwegian context, where the same domains (ie, domains 4 and 5) showed limited internal consistency [44,45].

### Sample Size

The a priori sample size calculation was based on the HLQ domain 9, “understanding health information well enough to know what to do,” and provided a change from baseline to 6-month follow-up, with an effect size of 0.5-unit difference between the arms on a scale of 1 to 5, with a 20% dropout, a power of 0.90, an SD of 0.6 from the outcome measure, and a 2-sided significance test. Thus, following a 1:2 recruitment ratio, the estimated required sample was 55 participants in the control arm and 110 participants in the intervention arm. The sample size estimate was split into 4 equally sized groups to estimate the number of participants needed to be recruited from each department. Originally, this 2-arm trial had an equal recruitment ratio across 4 departments, including the department of pain management, which planned to recruit patients with both chronic and acute postoperative pain [19]. However, recruitment of patients with postoperative pain ceased unexpectedly in March 2022, and the remaining recruitment was slow. To ensure the necessary statistical power and allow for more variation in the use of the intervention, our alterations prompted a new power analysis, and the follow-up period was reduced from 12 months to 6 months. Details of the power calculation and rationale for the effect size are summarized in the published protocol [19].

### Allocation and Blinding

This trial was a prospective, unblinded, nonrandomized controlled study. Allocation was conducted sequentially, meaning that patients were allocated to the control arm first, and when the required number was allocated, the participants were recruited to the intervention arm based on the sample size calculation. Allocation was conducted within each department, and when the required number of control participants were recruited, recruitment for the intervention arm began immediately, even if other departments were still recruiting for the control arm. Blinding was not possible because of the nature of the intervention.

### Ethical Considerations

Approval for the project was obtained after review by the institutional data protection officer at the University Hospital of North Norway for the cancer department (2021/4942) and by the data protection officer at the Oslo University Hospital for the remaining departments (21/06826). The project protocol was prereviewed by the regional ethics committee and regarded as outside the mandate according to the Norwegian Health Research Act (regional ethics committee southeast reference number 252051). All participants provided informed and written consent. All data were anonymized before analyses, and only the first author (HH) had access to the anonymizing key to safeguard participant information. Patients did not receive any compensation for their participation.

### Statistical Methods

All baseline data were reported as counts and percentages or means and SDs for normally distributed data and medians and ranges for skewed data. To investigate any baseline differences between the arms because of the lack of randomization, all baseline data were compared between the 2 arms using the chi-square test, Student *t* test (2-tailed), and Mann-Whitney *U* test, as appropriate. Missing data were not imputed. For all continuous variables, the individual mean change was estimated by subtracting the baseline score from the follow-up score at both 3 months and 6 months. Differences in mean changes between the arms were tested using the Student *t* test (2-tailed). A post hoc sensitivity analysis was performed using linear regression analysis, adjusting for the observed differences in the baseline characteristics between the arms (history of COVID-19 and domains 1 and 7 in eHLQ). *P*<.05 was considered statistically significant. Cronbach α was computed to evaluate the internal consistency of the scales based on the assumptions that the responses to individual questions were normally distributed, had equal variance, and equally explained the factor. All analyses were performed using SPSS (version 29; IBM Corp).

## Results

### Participant Flow

**Figure 1:**
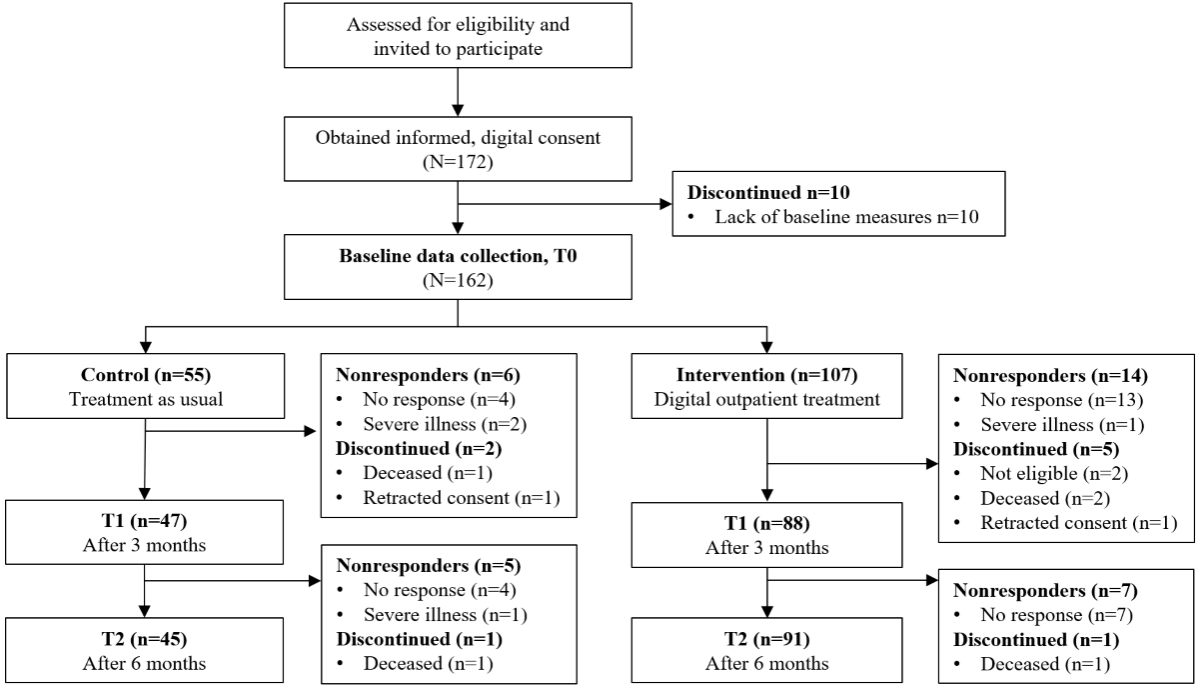
Flowchart of participation, from recruitment to follow-up. A total of 4 of the nonresponders at time point 1 (T1) in the control arm responded at time point 2 (T2), and 11 of the nonresponders at T1 in the intervention arm responded at T2. T0: time point 0.

During the recruitment period, 162 patients signed the digital informed consent forms and completed consecutive self-reported baseline questionnaires. Given the 1:2 allocation ratio in favor of the intervention arm, we successfully allocated 55 (34%) patients to the control arm and 107 (66%) patients to the intervention arm. The participant flow and discontinuation are depicted in Figure 1. Overall, recruitment and allocation to the control arm started in September 2021, and recruitment to the intervention arm started in January 2022; sufficient participants were recruited by December 2022 (specific dates of the start and end of the recruitment are provided in Multimedia Appendix 2). Recruitment to the 2 arms differed slightly among the 4 departments. The data collection ended in June 2023.

In the control arm, 11% (6/55) of the participants did not respond at 3 months, while among those still alive and included (ie, not with retracted consent) at 3 months, 10% (5/52) of the participants did not respond at 6 months. In the intervention arm, 13.1% (14/107) of the participants did not respond at 3 months, and 6.9% (7/101) of the participants did not respond at 6 months.

### Baseline Characteristics

The 162 participants included in this study, on average, were aged 52.6 (SD 15.8) years at baseline, with a range of 20 to 83 years (Table 2). There were 90 (56.6%) female participants; most (n=108, 66.7%) participants had a college education or higher, 99 (61.1%) were employed, and 32 (19.8%) were retired. There were no statistically significant differences between the control and intervention arms in terms of demographic or clinical characteristics, except for a history of COVID-19, subsequent fear of COVID-19 (Table 2), and a higher score in the intervention arm in eHLQ domains 1 and 7 (Table 3).

**Table 2:** Demographic and clinical characteristics at baseline (N=162).

Characteristics				Intervention arm (n=107), n (%)		Control arm (n=55), n (%)		*P* value^a^	
**Demographic characteristics**									
	Age (y; N=162), mean (SD)		51.8 (16.3)		54.1 (14.8)		.22		
	Age (y; N=162), median (range)		56.0 (20-83)		58.0 (22-78)		.29		
	Female sex (N=162), n (%)		60 (56.1)		30 (54.5)		.99		
	Education: college or more (N=162), n (%)		68 (63.6)		40 (72.7)		.32		
	**Employment status (n=161), n (%)**								.11
		Employed	70 (65.4)		29 (52.7)				
		Unemployed	15 (14)		15 (27.3)				
		Retired	21 (19.6)		11 (20)				
	Cohabitation status: cohabiting (N=162), n (%)		75 (70.1)		41 (74.5)		.68		
**Access to digital devices (N=162), n (%)**									
	Use of smartphone		106 (99.1)		54 (98.2)		.63		
	Weekly use of iPad		50 (46.7)		31 (56.4)		.32		
	Weekly use of PC		92 (86)		42 (76.4)		.19		
	Use of other health apps: yes		47 (43.9)		23 (41.8)		.80		
**Clinical characteristics**									
	**Diagnosis, n (%)**								.07
		Chronic pain (n=33)	21 (19.6)		12 (21.8)				
		Epilepsy (n=53)	40 (37.4)		13 (23.6)				
		Interstitial lung disease (n=39)	26 (24.3)		13 (23.6)				
		Cancer (n=34)	20 (18.7)		14 (25.5)				
		Postoperative pain^b^ (n=3)	0 (0)		3 (5.5)				
	Diagnosis duration (y; n=156), median (range)		5.0 (0-40)		5.0 (0-32)		.43		
	History of COVID-19 (n=160), n (%)		53 (49.5)		5 (9.1)		*<.001* ^c^		
	**Fear of COVID-19^d^ (n=102), n (%)**								*.03*
		Not afraid	25 (47.2)		11 (22.4)				
		A little afraid	24 (45.3)		30 (61.2)				
		Very afraid	4 (7.5)		8 (16.3)				
	Smoking or use of snuff: yes (n=161), n (%)		17 (15.9)		11 (20)		.52		
	Alcohol consumption: weekly or more (N=162), n (%)		58 (54.2)		32 (58.2)		.63		

^a^Between-arm differences were analyzed using the Student *t* test (2-tailed) and chi-square test for comparison of proportions as well as the Mann-Whitney *U* test for comparison of ranks (median).

^b^Recruitment of patients with postoperative pain was terminated before the required sample was allocated.

^c^Statistically significant values are italicized.

^d^Only those with no history of COVID-19 were asked about their fear.

**Table 3:** Health literacy, digital/eHealth literacy, and RAND-12 scores at baseline.

			Intervention arm (n=107), mean (SD)		Control arm (n=55), mean (SD)		*P* value
**Health literacy (N=162; 5 of the 9 domains of the HLQ^a^)**							
	Domain 1: feeling understood and supported by health care providers^b^	3.11 (0.52)		3.10 (0.47)		.91	
	Domain 2: having sufficient information to manage my own health^b^	2.90 (0.50)		2.88 (0.46)		.74	
	Domain 3: actively managing my own health^b^	2.85 (0.49)		3.01 (0.46)		.05	
	Domain 6: ability to actively engage with health care providers^c^	3.81 (0.72)		3.83 (0.59)		.85	
	Domain 9: understanding health information well enough to know what to do^c^	3.86 (0.55)		3.98 (0.43)		.16	
Digital/eHealth **literacy (N=162; 7 of the 7 domains of the eHLQ^d^)^e^**							
	Domain 1: using technology to process health information	2.89 (0.55)		2.70 (0.58)		*.046* ^f^	
	Domain 2: understanding of health concepts and language	2.97 (0.45)		3.00 (0.43)		.75	
	Domain 3: ability to actively engage with digital services	3.17 (0.51)		3.01 (0.58)		.06	
	Domain 4: feel safe and in control	3.15 (0.49)		3.23 (0.48)		.38	
	Domain 5: motivated to engage with digital services	2.92 (0.45)		2.81 (0.52)		.16	
	Domain 6: access to digital services that work	2.73 (0.46)		2.64 (0.44)		.19	
	Domain 7: digital services that suit individual needs	2.83 (0.49)		2.63 (0.55)		*.02*	
**RAND-12^g^ (n=161)**							
	Physical component score	39.2 (11.2)		40.2 (11.2)		.39	
	Mental component score	42.5 (10.4)		42.8 (10.4)		.76	

^a^HLQ: Health Literacy Questionnaire.

^b^Scored 1 to 4, where higher values indicate higher health literacy.

^c^Scored 1 to 5, where higher values indicate higher health literacy.

^d^eHLQ: eHealth Literacy Questionnaire.

^e^Scored 1 to 4, where higher values indicate higher eHealth literacy.

^f^Statistically significant values are italicized.

^g^Scored 0 to 100, where a higher standardized score indicates better health.

### Use of the Digital Service Intervention

The participants in the intervention arm used an Apple iOS device (52/106, 49.1%) more frequently than an Android operating system (31/106, 29.2%; missing information on system: 23/106, 21.7%). After 3 months, 75% (65/87) of the intervention arm responders self-reported still using the MyDignio app, and after 6 months, 8.6% (91/106) of the participants were categorized as high users. The use of the intervention was reported for the entire 6-month intervention (Table 4). Although patients from the cancer, lung, and neurology departments were expected to use the digital outpatient care throughout the 6 months they were in the trial, the pain department had patients in digital outpatient care for the purpose of both pain medication reduction and pain medication adjustments, typically for a shorter time than 6 months. Thus, patients from the pain department used the app for an average of 11.3 (SD 6.5) weeks, with a median of 9.5 (range 3-27) weeks.

**Table 4:** Interactions with the digital service during the 6 months of the study (n=106^a^).

Interactions				Intervention arm
**Digital interaction^b^**				
	**Digital interactions, n**			
		Mean (SD)	42.8 (52.3)	
		Median (range)	11.5 (0-265)	
	**Dichotomized digital interactions, n (%)**			
		Low use	15 (14.2)	
		High use	91 (85.8)	
**Asynchronous chat messages, n**				
	**Messages sent by patient**			
		Mean (SD)	6.1 (8.8)	
		Median (range)	2.0 (0-57)	
	**Messages sent by health care worker**			
		Mean (SD)	5.5 (6.6)	
		Median (range)	2.0 (0-27)	
	**Messages, n**			
		Mean (SD)	11.6 (14.7)	
		Median (range)	5.0 (0-76)	
**PRO^c^ measures, n**				
	**PRO measures sent to patient**			
		Mean (SD)	14.2 (18.8)	
		Median (range)	9.0 (0-120)	
	**PRO measure responses from patient**			
		Mean (SD)	11.8 (17.7)	
		Median (range)	4.0 (0-114)	
	**Monitoring (only patients with ILD^d^)**			
		Mean (SD)	79.0 (36.8)	
		Median (range)	76 (0-176)	

^a^n=1 missing.

^b^Computed value of the total number of chat messages, videos, completed patient-reported outcome measures, and monitoring.

^c^PRO: patient-reported outcome.

^d^ILD: interstitial lung disease.

### Changes After the Intervention

After 3 months, we observed a positive change in health literacy, which was measured using the HLQ (mean 0.08, SD 0.44) in the intervention arm, while a negative change (mean change –0.10, SD 0.51; *P*=.03) was observed in the control arm (Table 5). Similarly, in the HLQ domain 3, the intervention arm had a positive mean change compared with a negative mean change in the control arm. However, this change was not clinically significant (*P*<.50).

In HRQoL, a favorable mean change was observed in the intervention arm compared with the control arm for the physical component score (mean difference –3.29; *P*=.006) and mental component score (mean difference –3.08; *P*=.02).

For the primary outcome, health literacy after 6 months, which was measured using the HLQ domain 9, we did not observe any statistically significant differences in the scores (mean difference –0.05, 95% CI –0.20 to 0.10; *P*=.53; Table 6). Accounting for the differences in the history of COVID-19 and baseline eHLQ scores in domain 1 and 7 between the arms did not influence the results (mean difference –0.04; 95% CI –0.13 to 0.21, *P*=.66**)**. However, in the eHLQ, there was a statistically significant difference in domain 4 regarding feeling safe and in control, where the control arm had a positive change, while the intervention arm had a negative mean change.

**Table 5:** Mean scores and changes in health literacy, digital/eHealth literacy, and health-related quality of life (HRQoL) from baseline to 3 months (time point 1 [T1]).

Variables, domains, and arms				Baseline, mean (SD)		T1, mean (SD)		Change, mean (SD)
**Health literacy (5 of the 9 domains of the HLQ^a^)**								
	**Domain 1: feeling understood and supported by health care providers**							
		Intervention (n=88)	3.11 (0.52)		3.14 (0.51)		0.04 (0.49)	
		Control (n=47)	3.10 (0.47)		3.10 (0.47)		–0.02 (0.48)	
	**Domain 2: having sufficient information to manage my own health**							
		Intervention (n=88)	2.90 (0.50)		3.01 (0.45)		0.10 (0.40)	
		Control (n=47)	2.88 (0.46)		2.88 (0.54)		0.02 (0.46)	
	**Domain 3: actively managing my own health**							
		Intervention (n=88)	2.85 (0.49)		2.97 (0.48)		*0.13* ^b^ *(0.38)* ^c^	
		Control (n=47)	3.01 (0.46)		3.03 (0.47)		*0.02 (0.50)* ^c^	
	**Domain 6: ability to actively engage with health care providers**							
		Intervention (n=88)	3.81 (0.72)		3.86 (0.61)		0.02 (0.51)	
		Control (n=47)	3.83 (0.59)		3.73 (0.67)		–0.12 (0.51)	
	**Domain 9: understanding health information well enough to know what to do**							
		Intervention (n=88)	3.86 (0.55)		3.99 (0.46)		*0.08 (0.44)^d^*	
		Control (n=47)	3.98 (0.43)		3.90 (0.57)		–*0.10 (0.51)^d^*	
**Digital/eHealth literacy (7 of the 7 domains of the eHLQe)**								
	**Domain 1: using technology to process health information**							
		Intervention (n=87)	2.89 (0.55)		2.92 (0.47)		0.03 (0.44)	
		Control (n=46)	2.70 (0.58)		2.78 (0.58)		0.03 (0.37)	
	**Domain 2: understanding of health concepts and language**							
		Intervention (n=88)	2.97 (0.45)		3.04 (0.43)		0.06 (0.39)	
		Control (n=46)	3.00 (0.43)		3.04 (0.43)		0.00 (0.34)	
	**Domain 3: ability to actively engage with digital services**							
		Intervention (n=88)	3.17 (0.51)		3.19 (0.47)		0.02 (0.37)	
		Control (n=47)	3.01 (0.58)		3.03 (0.63)		–0.04 (0.28)	
	**Domain 4: feel safe and in control**							
		Intervention (n=88)	3.15 (0.49)		3.03 (0.49)		–0.10 (0.38)	
		Control (n=47)	3.23 (0.48)		3.13 (0.52)		–0.12 (0.44)	
	**Domain 5: motivated to engage with digital services**							
		Intervention (n=88)	2.92 (0.45)		2.88 (0.51)		–0.04 (0.46)	
		Control (n=47)	2.81 (0.52)		2.73 (0.52)		–0.10 (0.46)	
	**Domain 6: access to digital services that work**							
		Intervention (n=88)	2.73 (0.46)		2.73 (0.45)		0.01 (0.42)	
		Control (n=47)	2.64 (0.44)		2.61 (0.53)		–0.03 (0.43)	
	**Domain 7: digital services that suit individual needs**							
		Intervention (n=88)	2.83 (0.49)		2.88 (0.48)		0.08 (0.42)	
		Control (n=47)	2.63 (0.55)		2.59 (0.54)		–0.07 (0.52)	
**HRQoL**								
	**PCS^f^**							
		Intervention (n=90)	40.2 (11.1)		43.6 (11.7)		*1.73 (6.41)^g^*	
		Control (n=45)	39.2 (11.2)		38.0 (10.7)		–*1.57 (6.68)*^g^	
	**MCS^h^**							
		Intervention (n=90)	43.0 (10.4)		44.7 (11.1)		*1.10 (7.43)^i^*	
		Control (n=45)	42.5 (10.4)		41.2 (11.7)		–*1.98 (6.96)*^i^	

^a^HLQ: Health Literacy Questionnaire.

^b^Statistically significant values are italicized.

^c^The difference between the arms was in favor of the intervention arm (mean difference –0.15, 95% CI –0.30 to –0.00; *P*=.048).

^d^The difference between the arms was in favor of the intervention arm (mean difference –0.17, 95% CI –0.34 to –0.00; *P*=.03).

^e^eHLQ: eHealth Literacy Questionnaire.

^f^PCS: physical component score.

^g^PCS difference between the arms was in favor of the intervention arm (mean difference –3.29, 95% CI –5.62 to –0.96; *P*=.006).

^h^MCS: mental component score.

^i^MCS difference between the arms was in favor of the intervention arm (mean difference –3.08, 95% CI –5.64 to –0.52; *P*=.02).

**Table 6:** Mean scores and changes in health literacy, digital/eHealth literacy, and health-related quality of life (HRQoL) from baseline to 6 months (time point 2 [T2]).

Variables, domains, and arms				Baseline, mean (SD)		T2, mean (SD)		Change, mean (SD)
**Primary outcome: health literacy (domain 9 of the HLQ^a^)**								
	**Domain 9: understanding health information well enough to know what to do**							
		Intervention (n=91)	3.86 (0.55)		3.92 (0.49)		0.03 (0.44)	
		Control (n=45)	3.98 (0.43)		3.96 (0.54)		–0.02 (0.38)	
**Health literacy (4 of the 9 domains of the HLQ)**								
	**Domain 1: feeling understood and supported by health care providers**							
		Intervention (n=91)	3.11 (0.52)		3.10 (0.48)		–0.01 (0.47)	
		Control (n=45)	3.10 (0.47)		3.17 (0.49)		0.04 (0.49)	
	**Domain 2: having sufficient information to manage my own health**							
		Intervention (n=91)	2.90 (0.50)		3.01 (0.50)		0.08 (0.55)	
		Control (n=45)	2.88 (0.46)		2.93 (0.47)		0.05 (0.49)	
	**Domain 3: actively managing my own health**							
		Intervention (n=91)	2.85 (0.49)		2.96 (0.43)		0.11 (0.40)	
		Control (n=45)	3.01 (0.46)		3.01 (0.53)		–0.01 (0.51)	
	**Domain 6: ability to actively engage with health care providers**							
		Intervention (n=91)	3.81 (0.72)		3.87 (0.57)		0.02 (0.43)	
		Control (n=45)	3.83 (0.59)		3.73 (0.63)		–0.12 (0.52)	
**Digital/eHealth literacy (7 of the 7 domains of the eHLQ^b^)**								
	**Domain 1: using technology to process health information**							
		Intervention (n=91)	2.89 (0.55)		2.92 (0.49)		0.02 (0.41)	
		Control (n=45)	2.70 (0.58)		2.83 (0.63)		0.16 (0.44)	
	**Domain 2: understanding of health concepts and language**							
		Intervention (n=91)	2.97 (0.45)		3.01 (0.40)		0.03 (0.36)	
		Control (n=45)	3.00 (0.43)		3.10 (0.47)		0.12 (0.43)	
	**Domain 3: ability to actively engage with digital services**							
		Intervention (n=91)	3.17 (0.51)		3.14 (0.46)		–0.04 (0.38)	
		Control (n=45)	3.01 (0.58)		3.10 (0.63)		0.09 (0.45)	
	**Domain 4: feel safe and in control**							
		Intervention (n=91)	3.15 (0.49)		3.01 (0.42)		–*0.15*^c^ *(0.39)*^d^	
		Control (n=45)	3.23 (0.48)		3.25 (0.52)		*0.01 (0.46)* ^d^	
	**Domain 5: motivated to engage with digital services**							
		Intervention (n=91)	2.92 (0.45)		2.89 (0.51)		–0.03 (0.44)	
		Control (n=45)	2.81 (0.52)		2.81 (0.61)		0.00 (0.47)	
	**Domain 6: access to digital services that work**							
		Intervention (n=91)	2.73 (0.46)		2.27 (0.41)		–0.00 (0.47)	
		Control (n=45)	2.64 (0.44)		2.70 (0.55)		0.06 (0.51)	
	**Domain 7: digital services that suit individual needs**							
		Intervention (n=91)	2.83 (0.49)		2.88 (0.43)		0.04 (0.46)	
		Control (n=45)	2.63 (0.55)		2.70 (0.65)		0.09 (0.57)	
**HRQoL**								
	**PCS^e^**							
		Intervention (n=90)	40.8 (11.1)		43.6 (10.9)		2.10 (7.85)	
		Control (n=45)	39.2 (11.2)		39.2 (11.7)		–0.12 (6.95)	
	**MCS^f^**							
		Intervention (n=90)	43.0 (10.4)		45.2 (10.5)		1.79 (8.60)	
		Control (n=45)	42.5 (10.4)		44.3 (11.6)		0.82 (7.75)	

^a^HLQ: Health Literacy Questionnaire.

^b^eHLQ: eHealth Literacy Questionnaire.

^c^Statistically significant values are italicized.

^d^The difference between the arms was in favor of the control arm (mean difference 0.16, 95% CI 0.01-0.31; *P*=.046).

^e^PCS: physical component score.

^f^MCS: mental component score.

### Patient Satisfaction at 6-Month Follow-Up

There were no differences in general satisfaction with the outpatient service at 6-month follow-up, with 76% (34/45) of the participants in the control arm and 78% (71/91) in the intervention arm being satisfied (*P*=.42). There were no differences between the arms in terms of the perceived effect of the treatment on their own health; 18% (8/45) of those in the control arm reported a decline in their health compared with 14% (13/91) in the intervention arm, while the remaining participants reported an improvement or no change (*P*=.60). The proportion of participants stating that a digital outpatient service would affect their feeling of safety was 49% (27/55) of the control participants and 61.7% (66/107) of the intervention participants (*P*=.09).

The participants in the intervention arm were generally satisfied or had little concern about the digital intervention (Table 7). Technical difficulties were reported by 18.9% (20/106) of the participants at either the 3- or 6-month follow-up.

**Table 7:** Intervention arm satisfaction with the digital intervention at time point 2 (n=91).

SUTAQ^a^ domains^b^	Values, mean (SD)
Domain 1: enhanced care	5.10 (1.11)
Domain 2: increased accessibility	4.60 (1.17)
Domain 3: privacy and discomfort	2.99 (0.89)
Domain 4: care personnel concerns	3.27 (0.86)^c^
Domain 5: kit as a substitute	4.04 (0.94)^d^
Domain 6: satisfaction	5.35 (1.27)

^a^SUTAQ: Service User Technology Acceptability Questionnaire.

^b^For domains 1, 2, 5, and 6, a higher score indicates higher satisfaction, while domains 3 and 4 are reversed, where a higher score represents a concern.

^c^Cronbach α=0.62.

^d^Cronbach α=0.46.

### Use of Health Care Services

There were no differences between the 2 arms after 6 months in terms of medication changes or the number of times patients had contacted the outpatient clinic. The intervention arm reported significantly fewer hospital admissions than the control arm (*P*=.03). The intervention participants reported 37 hospital admissions (mean 0.35, SD 1.1; range 0-7) among 13 (12.1%) of 107 participants, while the control arm reported 25 hospital admissions (mean 0.5, SD 1.05; range 0-5) among 13 (26%) of 50 control participants.

## Discussion

### Principal Findings

This study reports findings from a study of a novel multicomponent digital health intervention comprising PRO measures, self-monitoring of physiological measures, and asynchronous chat messages among a heterogeneous group of patients in outpatient care. We did not observe any statistically significant differences in the HLQ domain 9, which assesses patients’ understanding of health information well enough to know what to do, even though this study was presumably adequately powered. Our results demonstrate a trend that digital outpatient care, at the 3-month follow-up, improved health literacy domains encompassing patients’ ability to actively manage their own health and whether they understood health information well enough to know what to do. In addition, both physical and mental HRQoL improved after 3 months of digital outpatient care compared with those in the control arm; however, this statistically significant change was not considered clinically significant (*P*<.50). At the 6-month term, these positive findings were not observed; in contrast, the patients in the intervention arm demonstrated a statistically significant decline in digital/eHealth literacy in the eHLQ domain *feel safe and in control*. Overall, the participants in digital outpatient care had a high satisfaction rate when evaluating the digital outpatient care platform. Previous research was inconclusive regarding the outcomes of digital health interventions [2-9]. Our study adds to this field by being the first study to explore the associated outcomes of a multicomponent and comprehensive digital outpatient intervention. Despite the noted limitations of our study, some reflections on our findings will be relevant to bring the research field forward.

Several mechanisms may explain our findings. Specifically, we argue that digital outpatient care is positively associated with health literacy and HRQoL because of several mechanisms of the digital care model. First, the use of digital outpatient care allows for the collection of PRO measures on a regular basis, thus increasing patient involvement. Favorable health outcomes related to such involvement have previously been observed [46], although evidence remains limited because of the heterogeneity of research studies in this field [9]. In our study, both the patients and the health care workers had access to a convenient tool for short, asynchronous messages through the digital platform. In line with previous research [47,48], for many of our patients, these asynchronous messages served as a tool to clarify minor questions, provide little information regarding their clinical status and self-management, and give a response without having to wait in a telephone queue. Finally, the department providing care for patients with ILD offered the patients equipment for home spirometry and pulse oximetry. Such home monitoring gave the patients their first experience of truly being able to self-manage their condition from home while data were sent to health care workers to ensure that proper care was provided.

Answering disease-related PRO measures can, in itself, increase reflection regarding self-management and skills in patients [20,49]. Favorable outcomes are also seen among those with the opportunity to ask questions regarding their own condition [47,48]. Altogether, enabling reflection and asking questions in the digital service might be the 2 crucial components supporting the short-term increase in health literacy in our study. Furthermore, limited health literacy has been suggested to predict the increased use of health resources [50]. Conversely, the observed increase in health literacy in our study might reduce the use of health resources over time, confirming previous research that found an association between supporting health literacy and improved self-management [9,14]. We found significantly fewer hospital admissions in the intervention arm, although this finding was not observed for the number of contacts with the outpatient clinic. The experience of increased self-management can also reduce the negative impact of living with a long-term or chronic condition, thus explaining the favorable change in HRQoL. However, as improved health literacy at 3 months was not found at 6 months, some uncertainty remains, and more research is needed to explore the association between health literacy and the use of health service resources.

Although the participants used digital tools to varying extents based on clinical indications, these new services provided favorable short-term outcomes. Short-term changes in favor of an intervention are often demonstrated, while long-term effects are more challenging to observe. Although we found an increase in health literacy and HRQoL after 3 months, we did not observe this increase after 6 months. Instead, we found a negative change in the digital/eHealth literacy domain concerning feelings of safety and control. We cannot fully explain these findings based on our data; however, we believe that the 6-month contact point represents a vulnerable time for patients in a digital outpatient care model. Initially, the new services spark curiosity, foster a sense of increased self-management, and help patients feel more capable of doing the right thing. However, after some time, this may change as patients develop their skills, use more digital care, and simultaneously become more aware of the elements they cannot control. Thus, while they might, in fact, have better control, their increased skills make them more cognizant of the uncertain factors, which creates the feeling of less control that we found. Another mechanism supporting this hypothesis is that despite the lower feeling of control, the participants reported high satisfaction with the digital service after 6 months. Previous research has not observed or discussed potential solutions to such issues. Thus, future research should be aware of the potentially increased need for support among patients during participation in a digital outpatient care model. This is not only to reduce dropout but also to reduce any potential feeling of losing control.

Patients with a persistent need for health services to support their self-management challenge traditional outpatient care, and new ways to provide patient care are warranted to uphold sustainable services of high quality [10]. Most of the participants in our intervention arm used digital outpatient care as intended; that is, they responded to PRO measures, uploaded self-monitoring measures, and asked for clarification when needed. These findings contrast with previous research, which suggests that maintaining participant engagement through a digital intervention can be challenging [51], even through a life course with chronic or long-term conditions [52,53]. Factors that are important for ensuring the use of digital care include a referral from a health care worker and an interest among both the patients and the health care workers in digital services [54]. In our study, we observed a low dropout rate and high satisfaction, suggesting that the patients were content with the study and the digital outpatient care they received. Whether the use and satisfaction of digital outpatient care would have been the case in a longer term beyond 6 months was beyond the scope of our study and should be investigated in future research. However, given how well the digital outpatient care was received and used by the patients, we suggest that models such as ours should be investigated further to explore their relevance and effects on clinical practice.

### Limitations

This study has some limitations, several of which were discussed in the published protocol [19]. First, there is a risk of selection bias because of the lack of randomization into either a control or intervention arm. However, notably, because the allocation was performed sequentially (first for the control arm and then for the intervention arm), there was no bias directly due to patient preferences. Moreover, the 2 arms were similar at baseline except for the history of COVID-19 and subsequent fear of COVID-19 and eHLQ domains 1, using technology to process health information, and 7, digital services that suit individual needs. The control arm was mainly recruited when very few people had contracted COVID-19, while the intervention arm was recruited when the strategy had shifted because of vaccinations and more people contracting COVID-19. As a result, the timeline of the COVID-19 pandemic and eHLQ may have caused some bias in this study. However, sensitivity analysis adjusted for baseline differences provided similar findings.

Second, we do not know how many patients were assessed for eligibility because these data were not sufficiently systematized during enrollment. We had to alter recruitment during the trial from a 1:1 allocation to a 1:2 allocation because of slow recruitment [19]. However, the revised power calculation with a sequential 1:2 allocation provided a sufficient sample size as anticipated a priori. This study was not powered to detect clinical differences within each of the 4 patient groups (ie, from the pain, lung, cancer, and epilepsy departments) or in any of the secondary objectives. Because of the many tests performed, false positive findings might have occurred, so the results should be interpreted with caution. Furthermore, we cannot provide evidence of the findings of digital outpatient care on disease-specific clinical parameters. Rather, we suggest that future research investigate whether our observed changes in health literacy affect disease-specific clinical or health outcomes over time, as has been observed in other studies [13,55].

### Conclusions

This study explored digital outpatient care comprising PRO measures; asynchronous messaging; and remote monitoring of clinical indications for patients with chronic pain, ILD, epilepsy, or cancer. No significant differences were observed between the 2 arms in our primary outcome, assessing patients’ health literacy reflected through their understanding of health information after 6 months. Our data indicate an improvement in some health literacy domains and HRQoL at 3 months among the patients using the digital solution. Despite our mixed results, the participants reported high satisfaction with the digital outpatient care intervention; therefore, digital health interventions can be positive for people with chronic conditions and are well received. However, our findings are not entirely consistent, and more research should explore the mixed changes in health or eHealth literacy, the effectiveness of such interventions on various outcomes, and the relationship between participant satisfaction and clinical effects.

## Appendix

Multimedia Appendix 1 Screenshots of MyDignio patient app and Dignio Prevent interface.

Multimedia Appendix 2 Specific recruitment dates.

## Abbreviations

eHLQ: eHealth Literacy Questionnaire
HLQ: Health Literacy Questionnaire
HRQoL: health-related quality of life
ILD: interstitial lung disease
PRO: patient-reported outcome
SPIRIT: Standard Protocol Items: Recommendations for Interventional Trial
STROBE: Strengthening the Reporting of Observational Studies in Epidemiology
SUTAQ: Service User Technology Acceptability Questionnaire

## Data Availability

The datasets generated or analyzed during this study are available from the corresponding author on reasonable request.
