# Supplementary material for: A Digital Outpatient Service With a Mobile App for Tailored Care and Health Literacy in Adults With Long-Term Health Service Needs: Multicenter Nonrandomized Controlled Trial
Source: J Med Internet Res. 2025 Apr 28;27:e60343. doi: 10.2196/60343 (PMC12070007; doi:10.2196/60343)
Supplement: Multimedia Appendix 1 [file jmir_v27i1e60343_app1.docx]

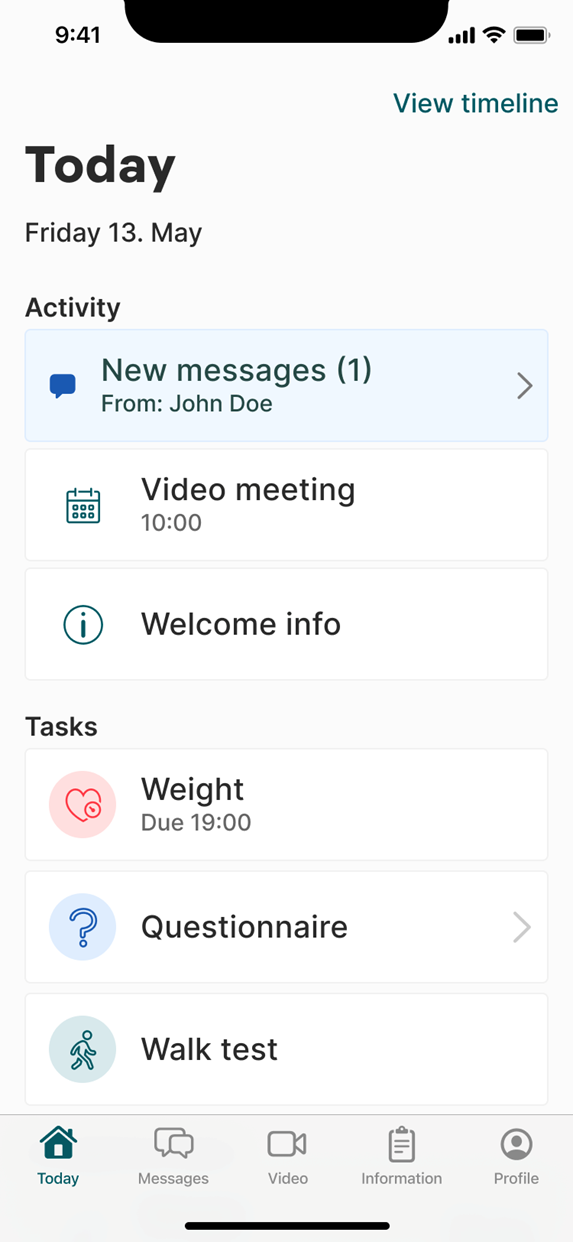


a) MyDignio. Screen of MyDignio, as the patients’ see it. All names, dates and values are reproduced and do not contain real patient data.


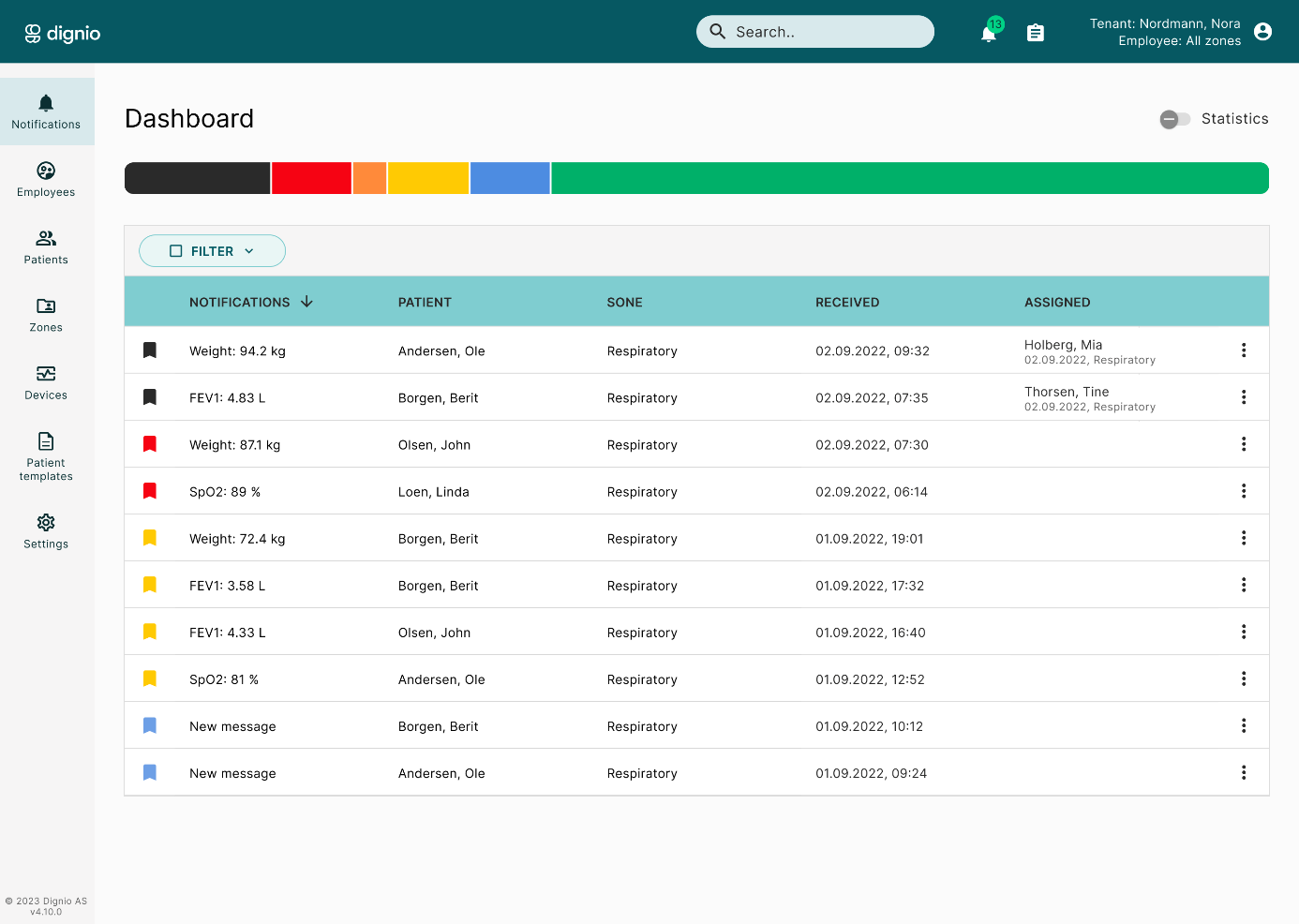


b) DignioPrevent. Dashboard as the healthcare workers see it. All names, dates and values are repoduced and do not contain real patient or healthcare worker data.
