# Supplementary material for: A Digital Outpatient Service With a Mobile App for Tailored Care and Health Literacy in Adults With Long-Term Health Service Needs: Multicenter Nonrandomized Controlled Trial
Source: J Med Internet Res. 2025 Apr 28;27:e60343. doi: 10.2196/60343 (PMC12070007; doi:10.2196/60343)
Supplement: Multimedia Appendix 2 [file jmir_v27i1e60343_app2.docx]

**Supplementary table 1. Specific dates of recruitment start and end**

| **Department** | **First participant in the control arm** | **Control arm completed** | **First participant in the intervention arm** | **Intervention arm completed** |
| --- | --- | --- | --- | --- |
| Neurology | September16th, 2021 | December 16^th^, 2021 | January 19^th^, 2022 | December 21^st^ 2022 |
| Respiratory Diseases | October 11th, 2021 | February 22^nd^ 2022 | March 8^th^ 2022 | December 22^nd^ 2022 |
| Pain Management and Research | September 12th, 2021 | January 17^th^ 2022 | February 8^th^ 2022 | December 14^th^ 2022 |
| Cancer | November 24th, 2021 | March 24^th^, 2022 | May 9^th^ 2022 | December 6^th^ 2022 |
